# Autophagy mediated by arginine depletion activation of the nutrient sensor GCN2 contributes to interferon-*γ*-induced malignant transformation of primary bovine mammary epithelial cells

**DOI:** 10.1038/cddiscovery.2015.65

**Published:** 2016-01-25

**Authors:** X-j Xia, Y-y Gao, J Zhang, L Wang, S Zhao, Y-y Che, C-j Ao, H-j Yang, J-q Wang, L-c Lei

**Affiliations:** 1 College of Veterinary Medicine, Jilin University, Changchun, PR China; 2 College of Animal Science, Jilin University, Changchun, PR China; 3 Changchun University of Chinese Medicine, Changchun, PR China; 4 College of Animal Science, Henan Institute of Science and Technology, Xinxiang, PR China; 5 College of Animal Science, Inner Mongolian Agricultural University, Hohhot, PR China; 6 College of Animal Science and Technology, China Agricultural University, Beijing, PR China; 7 Institute of Animal Science, Chinese Academy of Agricultural Science, Beijing, PR China

## Abstract

Autophagy has been linked to the regulation of both the prevention and progression of cancer. IFN-*γ* has been shown to induce autophagy in multiple cell lines *in vitro*. However, whether IFN-*γ* can induce autophagy and whether autophagy promotes malignant transformation in healthy lactating bovine mammary epithelial cells (BMECs) remain unclear. Here, we provide the first evidence of the correlation between IFN-*γ* treatment, autophagy and malignant transformation and of the mechanism underlying IFN-*γ*-induced autophagy and subsequent malignant transformation in primary BMECs. IFN-*γ* levels were significantly increased in cattle that received normal long-term dietary corn straw (CS) roughage supplementation. In addition, an increase in autophagy was clearly observed in the BMECs from the mammary tissue of cows expressing high levels of IFN-*γ*. *In vitro*, autophagy was clearly induced in primary BMECs by IFN-*γ* within 24 h. This induced autophagy could subsequently promote dramatic primary BMEC transformation. Furthermore, we found that IFN-*γ* promoted arginine depletion, activated the general control nonderepressible-2 kinase (GCN2) signalling pathway and resulted in an increase in autophagic flux and the amount of autophagy in BMECs. Overall, our findings are the first to demonstrate that arginine depletion and kinase GCN2 expression mediate IFN-*γ*-induced autophagy that may promote malignant progression and that immunometabolism, autophagy and cancer are strongly correlated. These results suggest new directions and paths for preventing and treating breast cancer in relation to diet.

## Introduction

Autophagy is an evolutionarily conserved catabolic process whereby cytoplasmic proteins and organelles are directed to lysosomes for degradation and recycling.^1^ Autophagy occurs at a low basal level under normal conditions to maintain cellular homoeostasis, but higher rates can be induced by many stimuli, including starvation and metabolic stress.^2,3^ In the mammary gland, autophagy has different functions that are important for homoeostasis in this organ, either under normal physiological conditions, such as contributing to the maintenance of cyclic remodelling, lactogenic hormones, sex steroids and cellular quality control, or in response to pathological stimuli, such as bacterial infections, toxic injuries or nutrient deprivation.^4,5^ Dysfunction in the autophagic pathway is implicated in a growing number of diseases, including breast cancer.^6,7^ Interferon gamma (IFN-*γ*) is a pleiotropic cytokine involved in a number of activities including, but not limited to, antiviral defence, host response during an infection, tumour immunosurveillance and control of cellular functions and replication.^8,9^ Emerging evidence suggests that IFN-*γ* signalling and autophagy interact.^10^

IFN-*γ* is primarily a proinflammatory cytokine produced by T cells and natural killer cells and is involved in the pathogenesis of the inflammatory disease multiple sclerosis (MS).^11,12^ In addition, IFN-*γ* exerts potent antiproliferative, antiangiogenic and immunomodulatory functions, which vary considerably among different cancer cell lines.^13,14^ Although IFN-*γ* has been used successfully to treat some solid cancers and haematological malignancies, it has displayed limited and transient effects in breast cancer patients.^15^ Furthermore, IFN-*γ* may, in some cases, even enhance cancer progression.^16^

What factors might induce the body to maintain high levels of IFN-*γ* long-term *in vivo*? An increasing number of studies have shown that diet affects immunity.^17,18^ G-protein-coupled receptors, which are metabolite sensors that are expressed in immune and intestinal epithelial cells, detect food metabolites or intestinal bacterial metabolites and then affect immune signalling pathways and alter cytokine levels.^19^ Malnutrition can lead to immune suppression. However, overnutrition can result in dysfunction of the immune response, chronic inflammation and cancer.^20,21^ IFN-*γ* is an inflammatory cytokine, and its levels are clearly influenced by diet.^22,23^ During lactation periods, under the influence of hormones, mammary tissue is highly metabolically active. Mammary epithelial cells proliferate, glands expand, duct secretion increases and tissue density increases. Thus, there is an increase in breast cancer risk factors during this period.^24,25^

IFN-*γ* secretion levels have recently been shown to be high due to stimulation from some dietary patterns.^22,23,26^ Therefore, human beings and other animals may easily have high levels of IFN-*γ* for long periods over the course of their life. IFN-*γ* has also been shown to induce autophagy in multiple cell lines and cancer cells *in vitro*.^27^ However, it is unknown whether IFN-*γ* can induce autophagy and whether autophagy promotes malignant transformation in healthy lactating bovine mammary epithelial cells (BMECs). In the present study, lactating Holstein cows, which have a long lactation period, were used as an experimental model and divided into two groups with the same dietary energy demands and the same concentrate component but different forage components in their feed. The levels of IFN-*γ* in serum and the autophagic activities in BMECs from mammary tissue *in vivo* were evaluated after 12 weeks. We found that the increased secretion of IFN-*γ* caused by the CS diet significantly enhanced autophagy of mammary epithelial cells *in vivo*. *In vitro*, we showed that IFN-*γ* can induce autophagy in healthy lactating BMECs through the activation of the GCN2 signalling pathway. Our findings are the first to demonstrate that arginine depletion and kinase GCN2 expression mediate IFN-*γ*-induced autophagy, that the ongoing activation of autophagy promotes BMEC transformation and that nutrition, immunometabolism, autophagy and cancer are strongly correlated. These findings may provide new insight for breast cancer prevention and therapy.

## Results

### A straw-based diet elevates IFN-*γ* levels

**Figure 1:**
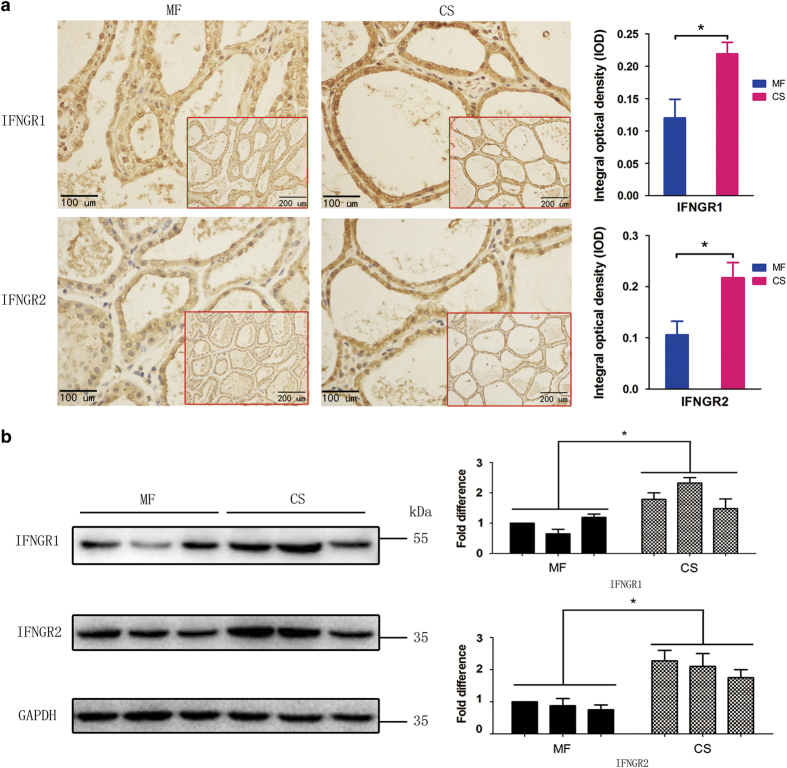
Expression of IFNGRs in cow mammary glands. Two groups of Holstein cows were fed with mixed forage (MF) or corn straw (CS). The experimental period was 12 weeks, and the pre-feeding period was 3 weeks. At the end of the feeding trial, the expression levels of IFNGR1 and IFNGR2 in mammary tissue (obtained via biopsy) were analysed using immunohistochemical staining and western blot analysis. (**a**) Immunohistochemical staining of IFNGR1 and IFNGR2. Scale bars, 100 *μ*m. Insets (scale bars, 200 *μ*m) show the overall presence of the brown colour indicating IFNGR1 and IFNGR2. Statistical analysis of the grey colour intensity (right). The data represent the mean±S.E.M. of three independent experiments. Error bars are±S.E.M. One-way ANOVA; *P<0.05. (**b**) Detection of IFNGRs via western blot analysis as described in the Materials and Methods section. The data represent the mean±S.E.M. of three independent experiments. Error bars are ±S.E.M. One-way ANOVA; *P<0.05.

In recent years, it has been shown that certain diets stimulate an increase in inflammatory cytokines in the body, increasing the risk of disease. First, we assessed whether a straw-based diet causes a change in inflammatory cytokine levels in cows. We used ELISAs to detect the concentration of IFN-*γ* in the serum of cattle. The average concentration of IFN-*γ* in the serum samples from the CS group was 481.84±22.21 ng/l, whereas that of the mixed forage (MF) group was 410.61±27.02 ng/l. This difference in IFN-*γ* concentration was significant (*P*<0.05). The same trend for IFN-*γ* levels was observed in milk samples, with the CS group (25.96 ng/l±6.95) having higher IFN-*γ* levels than the MF group (15.93 ng/l±5.58) (*P*<0.01). Consistent with the observed changes in animal serum and milk, the upregulation of IFNGR1 and IFNGR2 was also observed in paraffin-embedded and immunohistochemically stained sections of bovine mammary tissues (Figure 1a). Figure 1b shows that the relative protein expression levels of IFNGR1 and IFNGR2 were both increased in the CS group. Therefore, we concluded that a straw-based diet could induce an increase in IFN-*γ* levels *in vivo*.

### Straw-diet-driven IFN-*γ* enhances autophagic activity in mammary tissue epithelial cells

**Figure 2:**
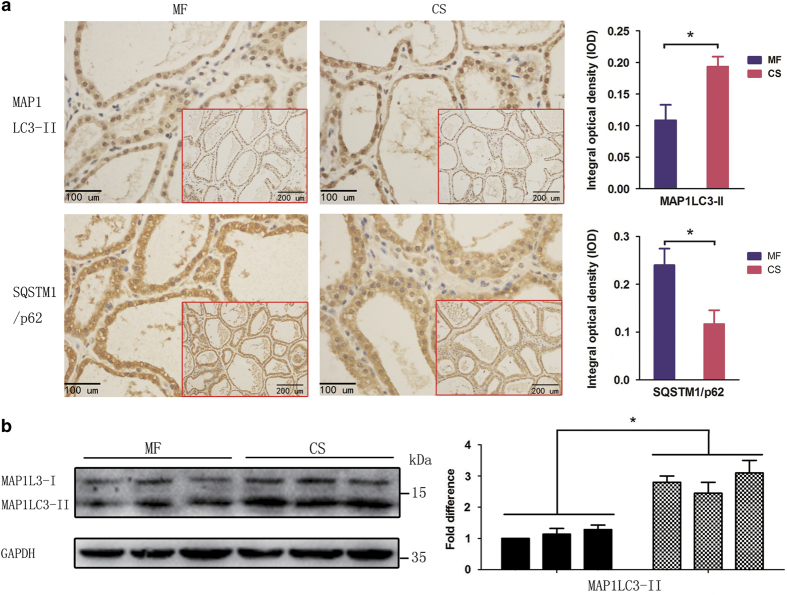
CS diet-dependent upregulation of autophagy in cow mammary glands. Two groups of Holstein cows were fed with mixed forage (MF) or corn straw (CS). At the end of the feeding trial, the levels of MAP1LC3-II and SQSTM1/p62 were analysed using immunohistochemical staining and western blot analysis. (**a**) Detection of MAP1LC3-II and SQSTM1/p62 using immunohistochemical staining. Scale bars, 100 *μ*m. Insets show the overall presence of the brown colour indicating MAP1LC3-II and SQSTM1/p62 (scale bars, 200 *μ*m). Statistical analysis of grey colour density (right). The data represent the mean±S.E.M. of three independent experiments. Error bars are ±S.E.M. One-way ANOVA; *P<0.05. (**b**) Detection of MAP1LC3-II and SQSTM1/p62 using western blot analysis as described in the Materials and Methods section. The data represent the mean±S.E.M. of three independent experiments. Error bars are ±S.E.M. One-way ANOVA; *P<0.05.

Autophagy participates in the physiological transition of mammary glands from lactating to non-lactating states and in the occurrence and development of breast cancer. We next examined whether the elevation of IFN-*γ* levels stimulated by a straw-based diet increased autophagy in BMECs. The levels of autophagic marker proteins, including MAP1LC3 (microtubule-associated protein 1 light chain 3) conversion and sequestosome 1 (SQSTM1/p62) degradation, were assessed in freshly isolated BMECs. After autophagy is initiated, proLC3 is processed, creating a form of LC3 that associates with the membrane and is known as LC3-II. The ratio of LC3-II to LC3-I indicates the level of cellular autophagy. SQSTM1/p62, which represents a link between LC3-II and ubiquitinated substrates, is degraded in the autolysosome, and its degradation marks the completion of autophagy. As shown in Figure 2a, we found significantly increased LC3-II expression and decreased SQSTM1/p62 expression in the CS group, compared with expression in the MF group. The integral optical density values of LC3B (microtubule-associated protein 1 light chain 3 beta) and SQSTM1/p62 were 0.193±0.028 and 0.117±0.083, respectively. Western blot analysis showed that LC3 was present primarily as LC3-II in mammary tissues from the CS group. In contrast, LC3 was present primarily as LC3-I in the MF group, with only a small amount of LC3-II (Figure 2b). Thus, LC3-II expression in mammary tissue biopsies from the CS group was significantly higher, whereas SQSTM1/p62 expression was significantly lower, compared with that in the MF group. These data indicate that autophagy was markedly enhanced in mammary tissues from the CS group. Thus, a straw-based diet induced high levels of IFN-*γ* production in the body, which correlated with an enhancement of autophagy.

### IFN-*γ* increased rates of autophagy in primary BMECs *in vitro*

**Figure 3:**
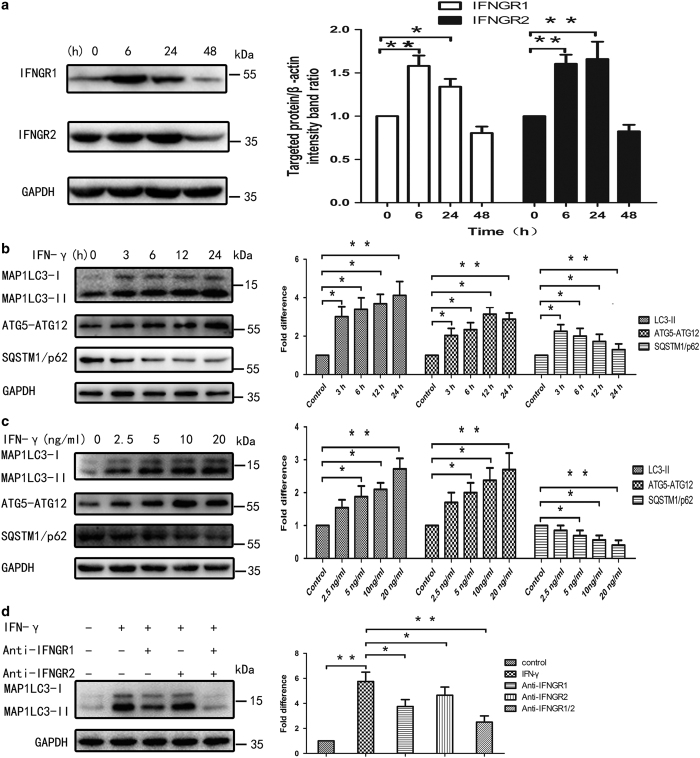
IFN-*γ* increased rates of autophagy in primary BMECs *in vitro*. (**a**) The expression of IFNGRs in BMECs. BMECs were treated with IFN-*γ* for 6, 24 or 48 h. At the end of the treatment period, the levels of IFNGR1 and IFNGR2 were analysed using western blot analysis with specific antibodies as described in the Materials and Methods section. The data represent the mean±S.E.M. of three independent experiments. Error bars are ±S.E.M. One-way ANOVA; *P<0.05; **P<0.01. (**b**) BMECs were treated with IFN-*γ* for 3, 6, 12 or 24 h. At the end of the treatment, levels of MAP1LC3, ATG12-ATG5, SQSTM1/p62 and GAPDH were analysed using western blot analysis with specific antibodies as described in the Materials and Methods section. The data represent the mean±S.E.M. of three independent experiments. Error bars are ±S.E.M. One-way ANOVA; *P<0.05; **P<0.01. (**c**) BMECs were treated with IFN-*γ* at 2.5, 5, 10 or 20 ng/ml. At the end of the treatment, levels of MAP1LC3, ATG12-ATG5, SQSTM1/p62 and GAPDH were analysed using western blot analysis with specific antibodies as described in the Materials and Methods section. The data represent the mean±S.E.M. of three independent experiments. Error bars are ±S.E.M. One-way ANOVA; *P<0.05; **P<0.01. (**d**) Direct effect of IFN-*γ* on induction of autophagy. BMECs were cultured for 24 h in supernatants from either mock-treated BMECs or cells treated with 10 ng/ml of IFN-*γ* for 24 h in the presence or absence of anti-IFNGR1, IFNGR2 or IFNGR1/2. At the end of the treatment, levels of MAP1LC3, ATG12-ATG5, SQSTM1/p62 and GAPDH were analysed using western blot analysis with specific antibodies as described in the Materials and Methods section. The data represent the mean±S.E.M. of three independent experiments. Error bars are ±S.E.M. One-way ANOVA; *P<0.05; **P<0.01.

To understand the relationship between IFN-*γ* and BMECs autophagy, we collected mammary tissues from healthy cows during lactation, isolated and identified BMECs and assessed induction of autophagy in primary BMECs exposed to IFN-*γ in vitro*. The biological effects of IFN-*γ* depended on the presence of IFN-*γ* receptors (IFNGRs) in the cell membrane. Western blot analyses revealed higher levels of IFNGR expression at 6 and 12 h than at 24 h after treatment with IFN-*γ* (Figure 3a). The data show that IFNGRs are expressed in BMECs and are likely to mediate the biological effects of IFN-*γ*. To determine whether IFN-*γ* induces autophagy, BMECs were treated with 10 ng/ml of IFN-*γ* for 6, 24 or 48 h. The results showed that the expression of LC3 gradually increased with time. The ratio of intracellular LC3-II to LC3-I proteins also significantly increased in a time-dependent manner (Figure 3b). Moreover, when cells were treated with 2.5, 5, 10 or 20 ng/ml of IFN-*γ* for 24 h, the ratio of LC3-II to LC3-I also significantly increased and exhibited an obvious concentration dependence (Figure 3c). ATG12-ATG5 is conjugated to form a ubiquitin-like protein-binding system that plays a key role in autophagic complex formation. We next examined the autophagic conjugation system by detecting the expression of ATG12-ATG5. The IFN-*γ*-treated cells had an increase in the formation of the ATG12-ATG5 complex relative to untreated cells in a time-dependent and dose-dependent manner (Figures 3b and c). After treatment with the anti-IFNGR antibody, the conversion of soluble MAP1LC3-I into MAP1LC3-II induced by IFN-*γ* was significantly disrupted (Figure 3d). These data indicate that IFN-*γ* induced autophagy specifically in BMECs *in vitro*.

As previously mentioned, SQSTM1/p62 correlates negatively with autophagic flux and its degradation marks the completion of autophagy. Western blot analysis showed that SQSTM1/p62 expression exhibited a decreasing trend in both a time- and dose-dependent manner (Figures 3b and c), suggesting that IFN-*γ* triggers a complete autophagic flux in BMECs.

**Figure 4:**
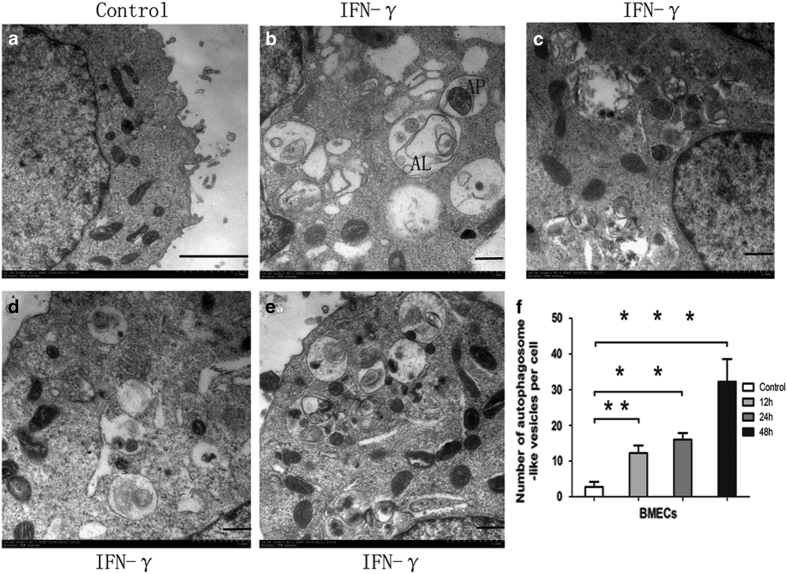
Transmission electron micrographs of IFN-*γ*-induced autophagy in BMECs. Cells were incubated with 10 ng/ml IFNA-*γ*. (**a**) Typical, untreated control cells and (**b**–**e**) representative cell populations 12 h (**b**, **c**), 24 h (**d**) and 48 h (**e**) after treatment. AP indicates autophagosomes, and AL indicates autophagolysosomes. (**f**) Quantification of the autophagosome-like vesicles per cell image. The average number of vesicles in each cell was obtained from at least 10 cells that were subjected to each treatment. Scale bar, 1 *μ*m. The data represent the mean±S.E.M. of three independent experiments. Error bars are ±S.E.M. One-way ANOVA; *P<0.05, **P<0.01, ***P<0.001.

Transmission electron microscopy (TEM) represents the gold standard for demonstrating autophagy. Using TEM, autophagosomes, which contain a large number of vacuoles enclosing cytolymph and organelles, can be observed in the cytoplasm. Early autophagosomes have a double-membrane structure containing undigested cytoplasmic constituents and have not yet fused with lysosomes. Late autolysosomes are identified by vesicles with a single-layer membrane that are formed by the fusion of autophagosomes and lysosomes and contain cytoplasmic components at various stages of digestion. To confirm that our observations of MAP1LC3 processing and ATG12-ATG5 complex formation were truly indicative of autophagy, we used TEM to investigate the effect of IFN-*γ* on BMECs. After BMECs were treated with IFN-*γ* for 12–48 h, more early autophagosomes and late autolysosomes were observed in the treated group than in the untreated group (Figures 4a and b). Moreover, induction of autophagy was time dependent (Figures 4b and f), indicating that IFN-*γ* functions upstream of autophagy and directly activates autophagy.

### Constant autophagy is required for IFN-*γ*-induced cell transformation in BMECs

**Figure 5:**
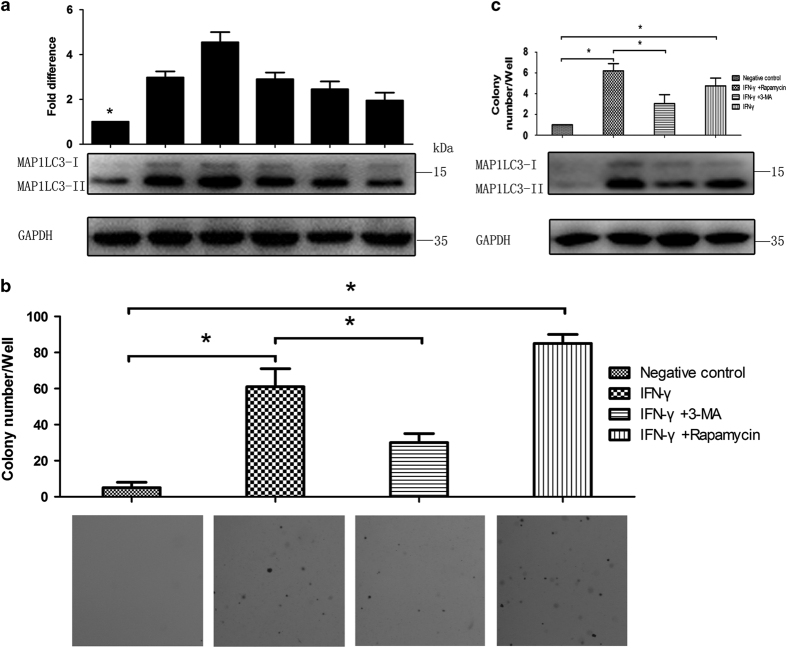
Autophagy is required for IFN-*γ*-induced BMEC transformation. (**a**) The expression levels of LC3-II in BMECs treated with IFN-*γ* (0 or 10 ng/ml) for up to 8 weeks were determined by western blot analysis with specific antibodies as described in the Materials and Methods section. The data represent the mean±S.E.M. of three independent experiments. Error bars are ±S.E.M. One-way ANOVA; *P<0.05; **P<0.01. (**b**) BMECs were exposed to IFN-*γ* (10 ng/ml), 3-MA (1 mM) or rapamycin (Rap; 10 nM) alone or in combination. Cells (1250) were grown on soft agar, and colonies were monitored after 3 weeks. The data represent the mean±S.E.M. of three independent experiments. Error bars are ±S.E.M. One-way ANOVA; *P<0.05, **P<0.01. (**c**) BMECs were exposed to IFN-*γ* (10 ng/ml), 3-MA (1 mM) or rapamycin (Rap; 10 nM) alone or in combination. The expression levels of LC3-II were analysed using western blot analysis with specific antibodies as described in the Materials and Methods section. The data represent the mean±S.E.M. of three independent experiments. Error bars are ±S.E.M. One-way ANOVA; *P<0.05; **P<0.01.

To determine the role of IFN-*γ*-induced autophagy in malignant transformation, we first determined whether autophagic activity was altered during IFN-*γ*-induced BMEC transformation. The cells were exposed to 10 ng/ml of sodium-IFN-*γ* for 8 weeks, as described in the Materials and Methods section. We first monitored autophagic activity by evaluating LC3-II expression. As shown in Figure 5a, IFN-*γ* treatment increased the expression level of LC3-II. After LC3-II upregulation reached a peak at 72 h, it decreased but remained at an elevated level, compared with the control, for 2–8 weeks in response to subchronic IFN-*γ* exposure. Cell transformation was determined by anchorage-independent growth in soft agar. After 8 weeks of treatment, IFN-*γ* induced more cell transformation than was observed in the control cells (Figure 5b). The cells were then either left untreated, treated with 3-methyladenine (3-MA) to inhibit autophagic flux, or treated with rapamycin to induce autophagy. The results demonstrated that 3-MA, an inhibitor of autophagy, reduced the IFN-*γ*-induced anchorage-independent cell growth on soft agar (Figure 5b). However, rapamycin produced the opposite effect, increasing IFN-*γ*-induced cell transformation (Figure 5b). Western blot analysis further showed that the level of LC3-II expression induced by IFN-*γ* was reduced in 3-MA-treated cells but was increased in rapamycin-treated cells compared with the levels observed in cells treated only with IFN-*γ* (Figure 5c). These data indicate that the transformation of BMECs are completely consistent with the intensity of autophagy. Taken together, these results indicate that the initiation of cell transformation was closely related to the autophagy induced by accumulated exposure to IFN-*γ*.

### IFN-*γ* induces autophagy in BMECs via a mechanism dependent on GCN2

**Figure 6:**
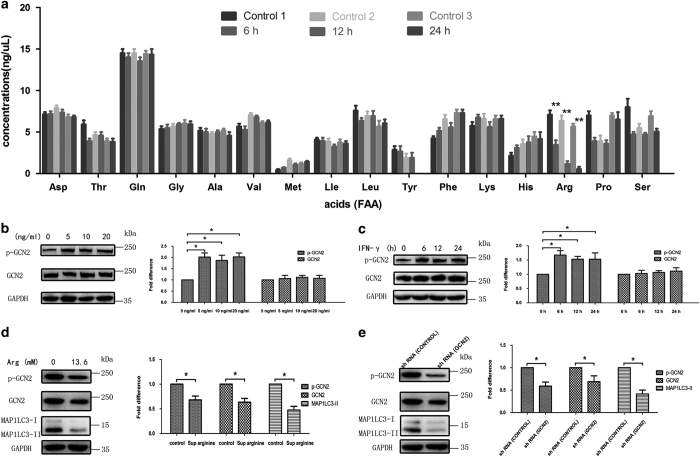
IFN-*γ* induces autophagy in BMECs via a mechanism dependent on GCN2. (**a**) Amino acids are metabolized during IFN-*γ* treatment. BMECs were incubated with 10 ng/ml IFN-*γ* for 6, 12 or 48 h or were left untreated. Sixteen types of free amino acids from the BMECs were detected by an amino acid analyser. The data represent the mean±S.E.M. of three independent experiments. Error bars are±S.E.M. One-way ANOVA; *P<0.05; **P<0.01. (**b**, **c**) BMECs were treated with different concentrations of IFN-*γ* (5, 10 or 20 ng/ml) for different times (6, 12 or 24 h). At the end of the treatment, the expression of p-GCN2, total GCN2 and GAPDH was analysed using western blot analysis with specific antibodies as described in the Materials and Methods section. The data represent the mean±S.E.M. of three independent experiments. Error bars are±S.E.M. One-way ANOVA; *P<0.05; **P<0.01. (**d**) BMECs were treated with IFN-*γ* (10 ng/ml). After 24 h, the cells were stimulated with arginine (13.6 mol/l) for 6 h. At the end of the treatment, the expression levels of GCN2, p-GCN2, MAP1LC3 and GAPDH were analysed using western blot analysis with specific antibodies as described in the Materials and Methods section. The data represent the mean±S.E.M. of three independent experiments. Error bars are ±S.E.M. One-way ANOVA; *P<0.05; **P<0.01. (**e**) Inhibition of GCN2 expression by adenovirus-delivered shRNA of GCN2. BMECs were infected with vAd-GFP-GCN2 or vAd-GFP-NT (NT represents the control shRNA). Twenty-four hours post-infection, total GCN2, p-GCN2, MAP1LC3 and GAPDH protein levels were analysed using western blot analysis with specific antibodies as described in the Materials and Methods section. The data represent the mean±S.E.M. of three independent experiments. Error bars are ±S.E.M. One-way ANOVA; *P<0.05; **P<0.01.

We next attempted to gain mechanistic insights into IFN-*γ*-induced autophagy in BMECs and hypothesized that amino acid deprivation is a master inducer of autophagy.^28^ Free amino acid analysis showed that culturing BMECs with IFN-*γ* resulted in a rapid decrease in the intracellular concentration of free arginine (Figure 6a). Decreased intracellular concentrations of free amino acids, including arginine, may lead to increased levels of uncharged transfer RNA molecules, which bind to GCN2 and induce its activation.^29,30^ Next, we tested whether IFN-*γ* could activate GCN2 kinase. As shown in Figures 6b and c, IFN-*γ* promoted GCN2 phosphorylation in a time- and dose-dependent manner. We also found that the phosphorylation of GNC2 was reversed by arginine supplementation (Figure 6c). Furthermore, arginine supplementation reduced LC3-II accumulation during IFN-*γ* exposure (Figure 6d). To address whether induction of autophagy in response to IFN-*γ* exposure was dependent on GCN2, we examined IFN-*γ*-induced autophagy in BMECs in which GCN2 expression was inhibited by an adenovirus-delivered shRNA of GCN2 (Figure 6e). The inhibition of GCN2 expression significantly reduced the formation of LC3-II in response to IFN-*γ* as assessed by western blotting analysis (Figure 6e), which suggests that the accumulation of autophagosomes in IFN-*γ*-treated BMECs depends on the presence of GCN2.

## Discussion

arcinogenesis of human breast epithelial cells is a multiyear, multistep, and multipath disease process, and more than 70% of human breast cancers are attributable to environmental factors such as exposure to chemical carcinogens and dietary habits.^31^ Autophagy is involved in several metabolic pathways, resulting in cytosolic material being targeted to the lysosomes for degradation and recycling, and plays an important role in the genesis and progression of tumours.^1,32^ In the present study, we found that a diet promoting IFN-*γ* secretion in the peripheral blood correlates with increased autophagic activity in BMECs in mammary tissue *in vivo*. Furthermore, we show that autophagy mediated by arginine depletion activation of the nutrient sensor GCN2 is involved in IFN-*γ*-induced malignant transformation *in vitro* and provide evidence that immunometabolism, autophagy and malignant transformation are strongly correlated.

The presence of several other cytokines and pathogens can also promote increased autophagy. Type I interferons have been reported to induce autophagy in many cell lines via the MTORC1 pathway, which is a key negative regulator of autophagy.^33^ Previous studies demonstrated the importance of Irgm1 in IFN-*γ*-induced autophagosome formation in RAW 264.7 cells.^34^ However, the human and bovine orthologue of Irgm1, IRGM, is not elicited by IFN-*γ*. Data regarding the regulation of autophagy in human and bovine healthy normal cells are very scarce. A recent study demonstrated that IFN-*γ* induces autophagy via JAK1/2 and p38 MAPK signalling but does not require STAT1.^35^ In the present study, we determined that a straw-based diet could induce high level of IFN-*γ* in lactating bovines, and we found evidence that IFN-*γ* may have autocrine effects on autophagy that are associated with a novel pathway used for GCN2 signalling *in vitro*. Our results could form the basis for the investigation of potential therapeutic targets in autophagic processes that may help to efficiently apply autophagy-regulating tumour therapies.

Thus far, studies investigating autophagy *in vivo* have been limited. Examples have been reported in cows, which experience autophagy in response to GH and IGF-1 late in the lactation period and in non-lactation periods, and in rainbow trout, in which muscle tissue autophagy follows high nutrient intake.^36^ To understand the role of autophagy in disease, studies investigating these process *in vivo* are necessary. Mammary gland tissue, which develops after the birth of an animal, experiences cycles of proliferation, branching, involution and remodelling, with further remodelling occurring during periods of lactation.^36^ Thus, mammary gland tissue is an ideal model in which to study the role of autophagy in the development of higher eukaryotes.^37^ During these processes, signals from stress and modelling induce changes in the matrix structure of the mammary tissue, resulting in cell death, polarization, growth retardation and autophagy.^38^ In daily life, diet is the most direct and important factor affecting the health of the body. Thus, studying mammary epithelial cell autophagy induced by dietary factors has real value for the prevention and treatment of breast cancer and other diseases.

Recent research has established that the level of IFN-*γ* is greatly influenced by diet. Regular intake of fermented rice bran increases the activity of IFN-*γ*. The intake of Brazil nuts significantly reduces serum IL-1, IL-6, TNF-α and IFN-*γ* levels, increases IL-10 levels and reduces inflammatory marker levels.^22,23,39,40^ Corn straw is a very common source of cow roughage in northern China. Corn stems and leaves are an abundant resource and may be stored conveniently. Many years of research have demonstrated that cows that are fed straw long-term have a higher risk of mammary disease and higher levels of IFN-*γ* in the blood and milk than cow that are not fed straw. The present work shows that the levels of IFN-*γ* stimulated by a straw diet are likely to induce autophagy in mammary epithelial cells. Our findings suggest that some diets may lead to non-infectious inflammation (sterile inflammation) in the body, which could promote a variety of diseases. Therefore, other cytokine disorders may be found to be linked to diet in future studies. Of course, food can also be used to modulate the immune system. Therefore, a balanced diet is important for maintaining health.^41^ Large animal models and many epidemiological surveys have shown that the intake of probiotics and fermented products can reduce the incidence of breast cancer.^42^

The mechanisms by which diet causes increased secretion of inflammatory cytokines remain unclear. Some studies suggest that the gut microbiota establishes a complex relationship with the host from birth, regulating the host’s metabolic pathways and altering the tolerance–inflammatory axis between the host and the microorganisms. This mutual relationship depends on the genome, nutrition and lifestyle of the host, and changes in diet can lead to changes in the gut microbiota and metabolites. Some nutrient receptors expressed by immune cells have been reported to activate the cells to produce different inflammatory cytokines.^43,44^ Some reports indicate that diets change the gut microflora, resulting in dysbacteriosis, increased LPS production and the release of proinflammatory cytokines, which can cause intestinal inflammation.^45^ In the present study, we showed the regulatory effect of a straw diet on inflammatory cytokines. The mechanism of action suggests new approaches for future research and may also aid in understanding the pathognesis of nutritional and metabolic diseases.

In conclusion, we have identified a new mechanism by which IFN-*γ* induces Arg metabolism. This then activates GCN2 kinase, which mediates IFN-*γ*-induced autophagy. Our study provides new insights into how autophagy regulates BMEC transformation *in vitro* and shows that immunometabolism, autophagy and cancer are strongly correlated, suggesting new directions and paths for preventing and treating breast cancer in relation to diet.

## Materials and Methods

The experiments were conducted in accordance with the National Legislation on Animal Care as certified by the Chinese Ministry of Agriculture.

### Antibodies and reagents

The main primary antibodies used in this study were specific for MAP1LC3 (Cell Signaling Technology, Beverly, MA, USA; Cat 2775), ATG5 (Novus Biologicals, Littleton, CO, USA; Cat NB110-53818), SQSTM1/p62 (Santa Cruz Biotechnology Inc., Heidelberg, Germany; Cat sc-25575), DAPDH (Cell Signaling Technology; Cat 2118), IFNGR1 (Bioss Biotechnology Co., LTD., Beijing, China; Cat bs-1463R), IFNGR2 (Bioss Biotechnology Co., LTD.; Cat bs-2710R), LC3B (Bioss Biotechnology Co., LTD; Cat bs-4843R) and SQSTM1 (Bioss Biotechnology Co., LTD; Cat bs-2951R). 3-Methyladenine and rapamycin were purchased from Santa Cruz Biotechnology Inc. 4′,6-diamidino-2-phenylindole (C1005) and pifithrin-*α* were purchased from Beyotime (Beyotime Institute of Biotechnology, Shanghai, China). HRP-conjugated goat anti-rabbit secondary antibodies were purchased from Proteintech (Proteintech Group, Inc., Chicago, IL, USA). Bovine interferon was purchased from the Kingfisher Group (Kingfisher Biotech, Inc., Saint Paul, MN, USA).

### Collection and analysis of milk and serum samples from experimental animals

Twenty multiparous (2–3 fetuses) Chinese Holstein cows were selected (DIM: 120±20 days, BW: 551±23 kg) and randomly divided into two groups. One group of 10 cows was fed an MF diet with a concentrate to roughage ratio of 45 : 55, containing Chinese wildrye, alfalfa hay and corn silage. The other 10 cows were fed a CS diet with the same concentrate to roughage ratio (45 : 55) as the MF diet. The CS diet contained CS as the only roughage material (Table 1). The entire experimental period lasted 12 weeks. The pre-feeding period was 3 weeks, and the official experimental period was 9 weeks. The final week was the sampling period. The cows were fed twice daily (0600 and 1800 hours) and milked twice daily (0800 and 1600 hours) under single-column type tethered feeding and free drinking conditions. Blood sampling was performed before feeding. Blood samples (10 ml) were collected from each cow. Serum was transferred to 1.5-ml tubes after separation and stored at −20 °C. Serum levels of IFN-*γ* were measured using an ELISA kit (Shanghai, China). Milk samples (100 ml) were collected in the morning, at noon and in the evening, and 0.1 g of potassium dichromate was added to each sample as a preservative. The milk samples were stored at 4 °C after collection, and the three milk samples obtained from each cow for each day were pooled before cytokine analysis.

### Immunohistochemical analysis

Three samples were collected from each group of cows using aseptic methods for *in vivo* mammary tissue sampling.^46^ Immunolocalization was conducted according to the literature.^47^ In brief, mammary gland samples were fixed in 10% neutral-buffered formalin, routinely processed (dehydration steps in ethanol and clearing in xylene) and embedded in paraffin blocks. Paraffin-embedded tissues were randomly cut into 4-mm sections using a microtome and placed on poly-l-lysine-coated glass slides. Immunohistochemical staining was performed using an avidin–biotin immunoperoxidase system. After the sections were incubated for 2 h at 40 °C, they were deparaffinized in xylene and hydrated through a graded ethanol series. Endogenous peroxidase activity was blocked by 3% H_2_O_2_ in 70% methanol. The sections were washed with PBS for 10 min (pH 7.3), and nonspecific protein-binding sites were blocked with 3% normal goat serum to reduce background staining. The sections were processed for immunolocalization of IFNGR1, IFNGR2, MAP1LC3-II and SQSTM1/p62 using commercially available polyclonal antibodies directed against IFNGR1 (1 : 100 dilution), IFNGR2 (1 : 100 dilution), MAP1LC3-II (1 : 100 dilution) and SQSTM1/p62 (1 : 100 dilution) as the primary antibodies. After the specimens were washed in PBS, they were incubated with biotinylated goat anti-polyvalent secondary antibodies for 30 min at room temperature. Streptomycin avidin-peroxidase conjugate was added, and specimens were incubated for 30 min at room temperature. The sections were visualized using 0.05% (w/v) 3,3′-diaminobenzidine and 0.010% (v/v) H_2_O_2_ in PBS (10 mM, pH 7.4). These sections were counterstained with haematoxylin and mounted with Entellan medium. Control procedures were performed to ensure the specificity of the immunoreaction. Negative controls were generated by replacing the primary antibodies with PBS. Images were acquired using an Olympus digital camera attached to an Olympus BX51 microscope (Olympus, Tokyo, Japan) . All of the slides were examined by the same observer, who was blind to which groups the tissue sections were from. Digital images were further quantitatively analysed using professional application software (Image-Pro Plus) to show the intensity of the staining.

### Cell culture and treatment

Mammary tissue samples were obtained from healthy cows during their lactation period using an aseptic method. BMECs were isolated and purified based on differences in endurance times for trypsin exposure and on the time required for adherence. Approximately 90% of BMECs was obtained in the fourth generation. To confirm the identity and purity of the BMECs, we initially performed morphological observations, growth curve assessments and karyotype analyses in addition to evaluations of other biological characteristics.^48,49^ Keratin-18 and vimentin antibodies were used to determine cell purity. qRT-PCR was used to detect the expression of the acetyl coenzyme A carboxylase *α* gene and the *β*-casein gene, which are important genes in the synthesis of milk components in BMECs. To observe the dynamic changes in IFN-*γ*-induced autophagy, BMECs were treated with 10 ng/ml of IFN-*γ* for 6, 24 or 48 h. To study the dose-dependence of IFN-*γ*, 2.5, 5, 10 or 20 ng/ml of IFN-*γ* was used to stimulate BMECs for 24 h. In the neutralization experiments, 10 ng/ml of anti-IFNGR1 and/or IFNGR2 antibody was applied to BMECs for 1 h before the addition of IFN-*γ* for 6, 24 or 48 h. Twenty-four hours after every subculture, the BMEC cultures were treated with 10 ng/ml of IFN-*γ* for 48 h, constituting one cycle of carcinogen exposure. The cultures were subcultured every 3 days (3 day/cycle), and the treatments were repeated for a total of 8 weeks.

### Western blot analysis

RIPA lysis buffer (Beyotime, Beyotime Institute of Biotechnology; Cat P0013B) containing protease inhibitors and phosphatase inhibitors was added to monolayer cells after the cells were washed twice in ice-cold PBS for 30 min. The lysate was centrifuged at 10 000×*g* at 4 °C for 10 min to remove insoluble material. The protein concentration was determined using the BCA method. Loading buffer was added to equal amounts of total cellular protein. The samples were denatured for 5 min at 100 °C, and 30 *μ*g of protein per sample was used for SDS-PAGE separation. The proteins that were separated via electrophoresis were transferred to PVDF membranes. Blocking was conducted for 4 h in 5% BSA at room temperature. Primary antibodies were added to the membranes and incubated overnight at 4 °C. The membranes were then washed five times (5 min per wash) in TBST. Luminescent fluid was prepared according to the manufacturer’s protocol, and protein expression was detected using the alpha chemiluminescent gel imaging system, FluorChem E. At the end of the treatment, the expression levels of target proteins were analysed with specific antibodies. The relative levels of the target proteins were estimated using densitometry, and the ratios were calculated relative to the GAPDH control.

### Adenoviral-delivered short hairpin RNA (shRNA) experiments

Tandem vAd-GFP-GCN2 adenovirus constructs (adenovirus-delivered shRNA of GCN2) and vAd-GFP-NT (non-template (NT) control shRNA) were purchased from Han Heng Biotechnology (Shanghai, China). For infection with the adenovirus constructs, BMECs were seeded in 24-well plates (the sterilized cell flyer was placed in the culture plates in advance) and cultured to 70% confluence in a DMEM/F12 medium containing 10% FBS at 37 °C in 5% CO_2_. After 24 h, the adenovirus construct was added to the cells at different MOIs at various time points. Then, the cells were gently mixed for 2 h at 37 °C. The culture medium containing the virus was removed and replaced with fresh medium containing IFN-*γ*. This medium was removed 24–48 h after infection, the cells were washed twice in PBS; then, 2.5% glutaraldehyde was added at 4 °C for 2 h for fixation, and the cells were washed three more times in PBS. Mounting medium was placed on the glass slides, and the cells were added. A laser confocal microscope (Olympus; FV300) was used to observe and image the cells.

### Transmission electron microscopy

To assess the occurrence of autophagy, a JEM-1200 EX TEM (JEOL) was used to observe the bilayer membrane vesicle structure of the autophagosomes. The cells were washed twice in PBS, placed in 1.5-ml EP tubes and then centrifuged at 1000×*g* for 5 min. The samples were fixed in 4% glutaraldehyde at 4 °C for 4 h. PBS buffer was used to wash the cells three times (10–45 min per wash), and 1% osmic acid was applied for fixation at 4 °C for 2 h. The cells were washed three times in distilled water (15 min per wash). The samples were then dehydrated by incubation in a solvent gradient of 50, 70, 80 and 95% ethanol (10 min each); a 1 : 1 mixture of 95% ethanol : 95% acetone for 10 min; 100% acetone twice for 20 min each; and propylene oxide once for 20 min. Propylene oxide and embedding medium (SPI-PON 812) were mixed 1 : 1 for 1 h of presoaking and were then used to saturate the embedding medium for 3 h. Polymerization was performed at 35 °C for 12 h, 45 °C for 12 h and 60 °C for 24 h. Ultra-thin sections were obtained and stained with uranyl acetate for 10 min, followed by a wash in distilled water, staining with lead citrate for 10 min and then a wash in distilled water. Finally, TEM was performed.

### Extraction of free amino acids from BMECs

After the BMECs were washed twice in ice-cold PBS, 1×10^7^ BMECs were digested with trypsin and harvested via centrifugation at 1000 r.p.m. for 10 min. The cell pellets were thoroughly degreased with lipase and organic solvent. Subsequently, the cells were washed in ice-cold PBS and disrupted via ultrasound. The protein present in the supernatant was precipitated using alcohol and filtered through a membrane with a 0.22-*μ*m pore size. The concentrations of 16 types of free amino acids in the BMEC supernatant were determined using an automatic amino acid analyser (L-8900; HITACHI, Tokyo, Japan).

### Soft agar assay

Cell transformation was determined by anchorage-independent growth in soft agar as stated in the manufacturer's instruction. Briefly, equal volumes of 0.8% agar and DMEM/F-12 were mixed at 40 °C. Then, 500 *μ*l of the mixture (containing 0.8% agar) was placed into 24-well plates as a base agar, and 1250 control or treated cells, suspended in 250 *μ*l of DMEM/F-12-agar mixture (containing 0.4% agar), were added in each well on top of the agar. The top of the agar was covered with 500 *μ*l of culture medium, which was replaced with fresh medium every 3 days. The plates were placed in an incubator at 37 °C and 5% CO_2_. After 4 weeks, the colonies were stained with 0.005% crystal violet and then quantified and photographed.

### Statistical analysis

Mean and S.E.M. values and sample sizes are indicated in each figure. The statistical analysis was performed with a one-way ANOVA using SPSS software (version 16.0). *P*-values <0.05 were considered significant; *P*<0.01, very significant; and *P*<0.001, extremely significant.

## Figures and Tables

**Table 1:** Composition and nutrient levels of experimental diets (DM basis, %)

*Item*	*Treatment (kg/day)*	
	*Mixed forage (MF)*	*Corn straw (CS)*
*Ingredient*		
Chinese wildrye	3.7	0.00
Alfalfa hay	23.40	0.00
Corn silage	26.70	0.00
Corn straw	0.00	53.80
Soybean meal	14.80	14.80
Cottonseed meal	5.10	5.10
Corn	24.60	24.60
Dicalcium phosphate	0.60	0.60
Salt	0.50	0.50
Dairy premix^a^	0.60	0.60
		
*Chemical composition*	*Nutritional level*	
NEL (Mcal/kg)	1.37	1.04
CP (%DM)	18.14	13.61
EE (%DM)	3.97	2.84
NDF (%DM)	38.57	47.02
ADF (%DM)	16.91	19.77
Ca (%DM)	0.82	1.06
P (%DM)	0.39	0.46
NFC (%DM)	28.39	25.88
NDF forage (%DM)	28.74	43.94
Starch (%DM)	25.50	15.32

a Dairy premix (DM): VA>70 IU; VD3>120 000 IU; VE>2100 mg; Fe 1750 mg; Cu 1600 mg; Zn l0 000 mg; Mn 3500 mg; Se 42 mg; I 84 mg; Co 42 mg.

## Abbreviations

BMECs: bovine mammary epithelial cells
MAP1LC3 (LC3): microtubule-associated protein 1 light chain 3
LC3B: microtubule-associated protein 1 light chain 3 beta
SQSTM1/p62: sequestosome 1 (a ubiquitin-binding scaffold protein)
TEM: transmission electron microscopy
MF: mixed forage
CS: corn straw
PI: propidium iodide
GFP-RFP-LC3: tandem GFP-RFP-LC3 adenovirus construct
GCN2: general control nonderepressible-2 kinase
3-MA: 3-methyladenine.
